# Cooperation between NRF-2 and YY-1 transcription factors is essential for triggering the expression of the *PREPL-C2ORF34 *bidirectional gene pair

**DOI:** 10.1186/1471-2199-10-67

**Published:** 2009-07-03

**Authors:** Chien-Chang Huang, Wun-Shaing Wayne Chang

**Affiliations:** 1 Institute of Life Sciences, National Defense Medical Center, Taipei 114, Taiwan; 2 National Institute of Cancer Research, National Health Research Institutes, Zhunan Town, Miaoli 350, Taiwan

## Abstract

**Background:**

Many mammalian genes are organized as bidirectional (head-to-head) gene pairs with the two genes separated only by less than 1 kb. The transcriptional regulation of these bidirectional gene pairs remains largely unclear, but a few studies have suggested that the two closely adjacent genes in divergent orientation can be co-regulated by a single transcription factor binding to a specific regulatory fragment. Here we report an evolutionarily conserved bidirectional gene pair, known as the *PREPL-C2ORF34 *gene pair, whose transcription relies on the synergic cooperation of two transcription factors binding to an intergenic bidirectional minimal promoter.

**Results:**

While *PREPL *is present primarily in brain and heart, *C2ORF34 *is ubiquitously and abundantly expressed in almost all tissues. Genomic analyses revealed that these two non-homologous genes are adjacent in a head-to-head configuration on human chromosome 2p21 and separated by only 405 bp. Within this short intergenic region, a 243-bp GC-rich segment was demonstrated to function as a bidirectional minimal promoter to initiate the transcription of both flanking genes. Two key transcription factors, NRF-2 and YY-1, were further identified to coordinately participate in driving both gene expressions in an additive manner. The functional cooperation between these two transcription factors, along with their genomic binding sites and some cis-acting repressive elements, are essential for the transcriptional activation and tissue distribution of the *PREPL-C2ORF34 *bidirectional gene pair.

**Conclusion:**

This study provides new insights into the complex transcriptional mechanism of a mammalian head-to-head gene pair which requires cooperative binding of multiple transcription factors to a bidirectional minimal promoter of the shared intergenic region.

## Background

In prokaryotic genomes, genes within operons usually have much shorter intergenic distances than genes at the borders of transcription units [1,2]. The distinct organization of operonic genes in near proximity is thought to facilitate gene co-expression and co-regulation. Recent evidence suggests that some eukaryotic genes also tend to be in close physical vicinity. For example, in the genomes of yeast, worm, and fly, a number of functionally related genes were shown to be located one near another [3-7]. In mammals, as genes are more dispersed throughout the genomes, the presence of two genes in immediate proximity of each other has long been regarded as a rare exception. Surprisingly, after the human and mouse genomes were decoded, there are more than 10% of the genes found to be arranged as bidirectional (head-to-head) gene pairs with two genes separated only by a less than 1 kb of genomic DNA [8-11]. Whilst some of these neighboring genes have evolved from a common ancestral gene by duplication, many are totally unrelated to each other and encode proteins involved in diverse biochemical pathways in different compartments of cells and tissues [10,12].

To date, the transcriptional regulation of mammalian head-to-head gene pairs remains largely unknown, but a few studies have suggested that the two closely adjacent genes in divergent orientation can be co-regulated by a single transcription factor binding to a specific promoter region [13-17]. Known examples include the human Parkinson's disease-related *PACRG-PARK2 *gene pair, the highly homologous mouse surfeit *Surf1-Surf2 *gene pair, and the non-homologous mouse *Sars2-Mrps12 *gene pair, in which the expression of two genes is delicately regulated by a transcription factor: in these cases N-myc, YY-1, and NF-Y, respectively [13-15]. In this report, we provide evidence for the existence of an evolutionarily conserved head-to-head gene pair, known as the *PREPL-C2ORF34 *gene pair, whose expression actually depends on the synergic cooperation of two transcription factors binding to a critical bidirectional regulatory element within a short intergenic region.

*PREPL *(prolyl endopeptidase-like), formerly named as *KIAA0436 *[18], is a member of the prolyloligopeptidase family of serine proteases [19,20]. Sequence analysis and secondary structure predictions revealed that this protease is similar to oligopeptidase B which utilizes a serine hydroxyl group to cleave target proteins. However, unlike oligopeptidase B which preferentially cleaves after an arginine or lysine residue, PREPL cannot cleave after any basic residue of the substrates [20]. This suggests that the function of PREPL may be different from that of oligopeptidase B and other known prolyloligopeptidases [20]. Clinical studies have demonstrated that chromosomal deletions involving the *PREPL *gene often lead to autosomal recessive syndromes such as hypotonia, facial dysmorphism, neonatal seizures and developmental delay [21,22]. The severity of these syndromes is closely related to the size of chromosomal deletion and the number of genes involved. For instance, patients with a relative mild HCS (hypotonia-cystinuria syndrome) are missing both alleles of *SLC3A1 *(solute carrier family 3, member 1) and *PREPL *on chromosome 2p21. In a more severe, atypical HCS phenotype, an additional gene *C2ORF34 *(chromosome 2 open reading frame 34) is deleted, whereas in the most severe 2p21 deletion syndrome the open reading frame of *PPM1B *(protein phosphatase magnesium-dependent 1B) is seriously disrupted [19,22-26]. Interestingly, among these four cluster genes that are associated with autosomal recessive disorders, only *PREPL *and *C2ORF34 *were found to lie immediately adjacent to each other in a head-to-head configuration in many species including human, mouse, rat, dog and chicken. The evolutionarily conserved close proximity and divergent orientation indicate that the two genes are likely co-regulated by some shared intergenic regulatory elements.

Herein, we determined that *PREPL *and *C2ORF34 *are separated by only a 405-bp intergenic DNA region in the human genome. Within this small intergenic region, a 243-bp GC-rich segment was shown to function as a bidirectional minimal promoter to initiate transcription of both flanking genes. By performing a series of promoter analyses, including chromatin immunoprecipitation and electrophoretic mobility shift assays, two transcription factors, NRF-2 and YY-1, were indentified to be involved in the regulation of the bidirectional minimal promoter. The functional cooperation between these two key transcription factors, along with their genomic binding sites and some cis-acting repressive elements, is essential for the transcriptional regulation and tissue distribution of the *PREPL-C2ORF34 *head-to-head gene pair.

## Results

### Tissue expression patterns of human PREPL and C2ORF34 genes

**Figure 1:**
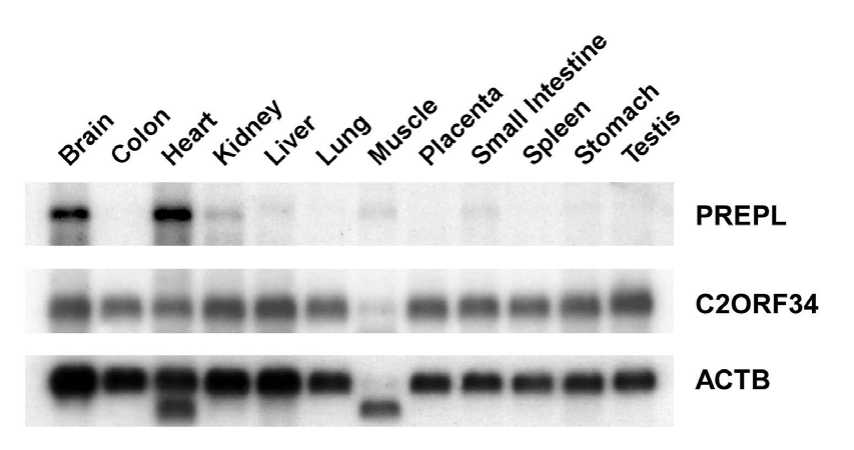
**Northern blot analysis of *PREPL *and *C2ORF34 *in various tissues**. Human 12 major tissue polyA+ RNA blot (OriGene) was hybridized with ^32^P-labeled *PREPL *(upper panel), *C2ORF34 *(middle panel) and control *ACTB *(β-actin; lower panel) cDNA probes. Note that the commercial *ACTB *probe can recognize non-muscle β-actin (upper band) as well as smaller size α- or γ-actin isoform (lower band) found in certain tissues such as heart and muscle.

In order to determine the presence and expression patterns of the *PREPL *and *C2ORF34 *genes, we performed Northern blot analysis on polyA+ RNA isolated from 12 different human tissues. As illustrated in Figure 1, while the *PREPL *probe identified a band of ~5 kb present predominantly in brain and heart, hybridization of the same array with the *C2ORF34 *probe revealed an abundant band of ~2 kb detected in almost every tissue examined. This suggests that the transcriptions of the *PREPL *and *C2ORF34 *genes in most tissues are likely to be differentially controlled by some regulatory elements situated within their 5'-upstream intergenic region.

### Determination of the size of the intergenic region between human C2ORF34 and PREPL genes

**Figure 2:**
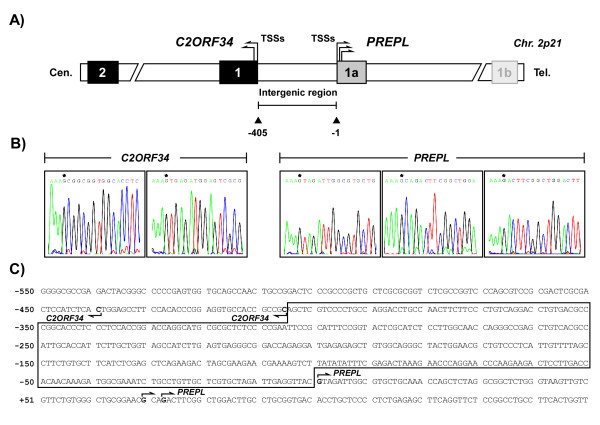
**Determination of the size and sequence of the intergenic region between human *C2ORF34 *and *PREPL *genes**. A) The first few exons of *C2ORF34 *(black boxes) and *PREPL *(grey boxes), the introns, and the shared intergenic segment (middle white bar) are shown. Bent arrows represent the gene directions and transcriptional start sites (TSSs) identified by 5'-RACE. The shortest intergenic distance between the two genes is calculated to be 405 bp and numbered (from -1 to -405) from the telomeric (tel) to the centromeric (cen) end. B) The 5'-RACE products, which were sequenced from the shared intergenic region to the direction of downstream gene transcription, revealed multiple TSSs (marked with an asterisk) including two for *C2ORF34 *and three for *PREPL*. C) The bent arrows and the bold C and G nucleotides denote the different TSSs for *C2ORF34 *and *PREPL*, respectively. The 405-bp intergenic region is boxed.

**Figure 3:**
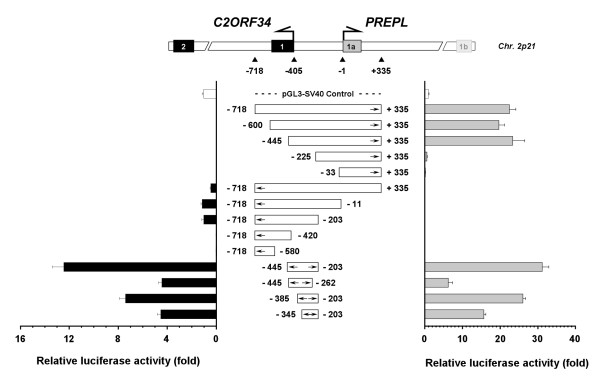
**Determination of the promoter activity of the intergenic region**. The directions of gene transcription are indicated by arrows. Here, a 1053-bp fragment (from position -718 to +335) consisting of the entire 405-bp intergenic region and ~300 bp beyond its two boundaries is cloned as the full-length promoter construct. Progressive deletions from this full-length promoter construct were produced and inserted into the promoterless vector in either the sense (relative to *PREPL*) or antisense (relative to *C2ORF34*) direction. The resulting luciferase activity of transiently transfected U87MG cells is given adjacent to each construct. Co-transfection of pRL-TK plasmid was performed for normalization of transfection efficiencies and cell viability. Units are relative to SV40 control promoter, defined as 1×. The experiments were performed in triplicate. Data are representative of three independent experiments, and error bars are ± standard deviation. A similar result was also observed in human H4 cells (data not shown).

Following characterization of tissue distribution patterns, we attempted to pinpoint the precise transcriptional start sites (TSSs) of the *C2ORF34 *and *PREPL *genes to ascertain the length of their intergenic spacer. We first utilized exon-specific primers to amplify genomic DNA fragments encompassing the first exons of both genes. The PCR products were then purified and sequenced to confirm that these two genes are indeed arranged in a head-to-head configuration on human chromosome 2p21 (Figure 2). By performing 5'-RACE with fetal brain-derived cDNA as a template, both genes were identified to contain multiple TSSs including two for *C2ORF34 *and three for *PREPL *(Figure 2B). It should be noted that, during the course of 5'-RACE analysis, we unexpectedly detected a *PREPL *splice variant which uses an alternative, 184-bp untranslated exon 1 (denoted as exon 1a, see Figures 2, Figure 3, and Additional File 1). With this newly found 5'-untranslated exon, the shortest intergenic distance between the closest TSSs for *PREPL *and *C2ORF34 *was calculated to be 405 bp (Figure 2). In order to facilitate our subsequent analyses of the promoter activity of this 405-bp intergenic region, here we denoted the positions of nucleotides with respect to -1, the first intergenic nucleotide in front of the *PREPL *transcriptional start site, and -405, the intergenic nucleotide right before the transcriptional start site of *C2ORF34 *(see Figure 2).

### Interspecies comparison of the gene order, orientation and intergenic spacing of the PREPL and C2ORF34 genes

To investigate whether the close physical proximity and head-to-head configuration of the *PREPL *and *C2ORF34 *genes are conserved throughout evolution, we compared the chromosomal localization and transcriptional orientation of both genes in different species including human, mouse, rat, dog and chicken. The results revealed that not only the gene order but also the compact head-to-head arrangement are evolutionarily conserved (data not shown). Even the size and nucleotide sequence of their intergenic spacing were found to be conserved, with a fragment of about 300 bp spanning from part of the exon 1 of *C2ORF34 *to the first two-thirds of the intergenic region, showing an average GC content greater than 70% (see Additional File 2). By aligning the human, mouse and rat sequences of this well-conserved, GC-rich region, several consensus transcription factor binding sites were identified. On the basis of these findings, we then attempted to analyze the possible role of these sites in governing the expression of the *PREPL-C2ORF34 *bidirectional gene pair.

### Functional characterization of the asymmetric bidirectional promoter activity of the intergenic region

In order to identify the key elements responsible for the transcriptional regulation of the *PREPL-C2ORF34 *gene pair, a series of DNA fragments containing all or part of the 405-bp intergenic region were cloned into the promoterless pGL3-basic vector followed by luciferase activity assays. Due to the fact that the regulatory elements are often situated within 300-bp upstream or downstream of the TSSs [27,28], here we cloned a 1,053-bp fragment, from position -718 to +335 (see Figure 3), consisting of the entire intergenic region and approximately 300 bp beyond its two boundaries, as the full-length promoter construct. By measuring the luciferase activity in human U87MG cells where both *C2ORF34 *and *PREPL *were adequately expressed, this full-length -718/+335 construct was shown to possess a promoter activity stronger than the control SV40 in driving reporter gene expression in the sense (relative to *PREPL*) orientation (Figure 3). Several progressive deletions from the 5'-end of the full-length -718/+335 construct were then generated in an attempt to determine the minimal promoter region necessary to drive *PREPL *gene transcription. As summarized in Figure 3, results from luciferase activity measurements pinpointed a 220-bp segment, from -445 to -226 spanning the first 40 bp of *C2ORF34 *exon 1 to the 180-bp 5'-end of the intergenic region, that is critical for the transcription of the *PREPL *gene. Similar progressive deletions from the full-length +335/-718 insert were also constructed to clarify the promoter activity in the antisense (relative to *C2ORF34*) direction. The luciferase assays demonstrated that a 218-bp segment, from -203 to -419 spanning the 5'-end 203-bp of the intergenic region and the first 14 bp of *C2ORF34 *exon 1, is responsible for driving transcription of the *C2ORF34 *gene (Figure 3).

Based on the above results, we then cloned a 243-bp fragment (from position -445 to -203, see the first "← →" bar in Figure 3) covering both the identified -445/-226 promoter region for *PREPL *and the -203/-419 promoter region for *C2ORF34 *in two opposite orientations and measured its potential bidirectional promoter activity. To our surprise, while no obvious difference was observed between the assays driven by the 243-bp (-445/-203) fragment alone or by the full-length (-718/+335) construct toward the *PREPL *direction, the same 243-bp fragment but in opposite orientation (-203/-445) was able to significantly increase the luciferase activity in the *C2ORF34 *direction by at least 12-fold as compared to the -203/-718 or the control +335/-718 full-length insert (Figure 3). Furthermore, removal of the sequence of the 243-bp fragment either from the 5'- or 3'-end (see the last three "← →" bars in Figure 3) all resulted in a decrease of the luciferase activities in both directions. Together these findings suggest that the 243-bp fragment is likely to function as a bidirectional minimal promoter, and that the region from position -446 to -718 may serve as a repressive element to suppress the transcription activity of the *C2ORF34 *gene.

**Figure 4:**
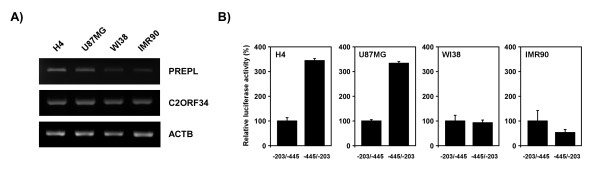
**Effect of promoter strength of the 243-bp regulatory element on the expressions of *C2ORF34 *and *PREPL *genes**. A) Semi-quantitative RT-PCR analysis of *C2ORF34*, *PREPL*, and control *ACTB *transcripts. RNAs used in this analysis were obtained from human brain (H4 and U87MG) and lung (WI38 and IMR90) cells. The specific primers for each gene are indicated in the methods. B) The relative luciferase activities driven by the 243-bp (-445/-203) fragment toward either the *C2ORF34 *or *PREPL *direction in different cells were determined. The transfection efficiency and cell viability were normalized with the pRL-TK plasmid. Units are relative to the luciferase activity driven by the 243-bp bidirectional minimal promoter in *C2ORF34 *direction, defined as 100%. The experiments were performed in triplicate and repeated independently at least twice. The error bars are ± standard deviation.

To further confirm the functional significance of the identified 243-bp regulatory element, we determined the effect of its promoter strength on the expressions of the two flanking genes. The results, similar to those obtained by the aforementioned Northern blot analyses (Figure 1), revealed that the levels of *C2ORF34 *transcripts remained constant in various cell lines whereas *PREPL *was found to be expressed in brain H4 and U87MG cells but much less in lung WI38 and IMR90 cells (Figure 4). In accordance with this, although no notable difference was observed in the relative luciferase activity driven by the 243-bp (-445/-203) fragment toward the *C2ORF34 *direction, the relative luciferase activity of the same fragment toward the opposite *PREPL *direction was shown to be more elevated in brain than in lung cells (Figure 4). These results indicate that the 243-bp minimal promoter element is likely responsible for directing differential expression of the *PREPL *gene, if not also for the neighboring *C2ORF34 *gene.

It has previously been proposed that, in addition to the bidirectional minimal promoter, DNA methylation may also be participated in the transcriptional regulation of bidirectional gene pairs [29,30]. By using Methprimer software  and a modified criteria [31], which defined the size of a CpG island as larger than 200 bp and the ratio of observed versus expected CpGs as greater than 0.7, we identified a GC-rich fragment spanning from part of the exon 1 of *C2ORF34 *to the first two-thirds of the 405-bp intergenic region (see Additional File 2). To elucidate whether the differential expression of *C2ORF34 *and *PREPL *is contributed by DNA methylation, we measured the methylation status of each individual CpG site. The assay results displayed no notable differences in the CpG methylation patterns of various normal tissues (Additional File 2). This indicates that the tissue-specific pattern of both *C2ORF34 *and *PREPL *genes may not be controlled directly by DNA methylation, but rather be regulated by some transcriptional factors binding to the identified 243-bp bidirectional minimal promoter.

### Identification of transcription factor binding sites in the bidirectional minimal promoter

**Figure 5:**
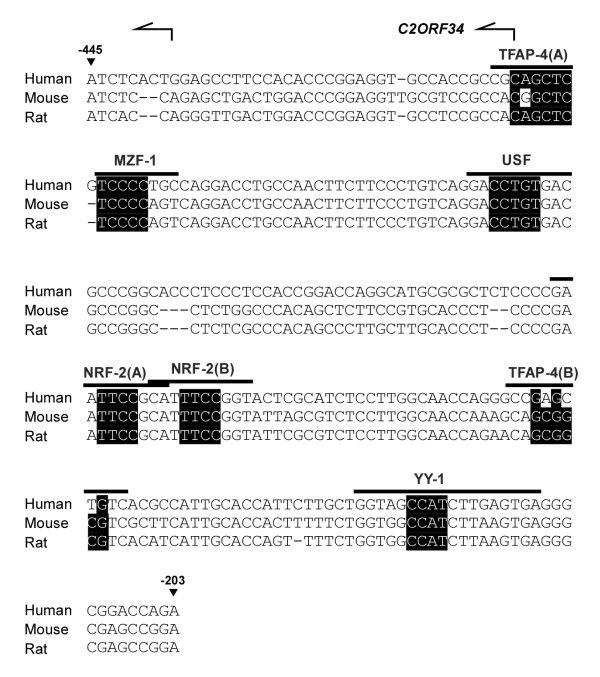
**Multiple sequence alignment of the bidirectional minimal promoter region of the *PREPL-C2ORF34 *gene pair**. The identified 243-bp bidirectional minimal promoter region on human chromosome 2p21 spans from the first 40-bp (position -445 to -406) of *C2ORF34 *exon 1 to the first half (position -405 to -203) of the intergenic region. All putative transcription factor binding sites found in this bidirectional promoter are indicated above the alignment. The core sequence of each transcription factor target site is highlighted in black, with the bent arrows representing two *C2ORF34 *TSSs.

**Figure 6:**
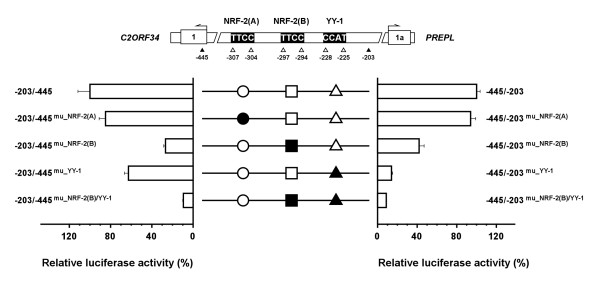
**Identification of the functional significance of the NRF-2 and YY-1 binding sites in the bidirectional minimal promoter**. Mutations at the first NRF-2 binding site are shown as a filled circle and denoted as -203/-445^mu_NRF-2(A) ^in the antisense (relative to *C2ORF34*) and as -445/-203^mu_NRF-2(A) ^in the sense (relative to *PREPL*) directions. Mutations at the second NRF-2 binding site are shown as a filled square and named as -203/-445^mu_NRF-2(B) ^in the antisense and as -445/-203^mu_NRF-2(B) ^in the sense directions. Mutations at the YY-1 binding site are shown as a filled triangle and termed as -203/-445^mu_YY-1 ^and -445/-203^mu_YY-1 ^in two different directions. Double mutations at both the NRF-2(B) and the YY-1 binding sites are denoted as -445/-203^mu_NRF-2(B)/YY-1 ^and -203/-445^mu_NRF-2(B)/YY-1^. Units are relative to the wild-type bidirectional minimal promoter, defined as 100%. The experiments were performed in triplicate. Data are representative of three independent experiments, and error bars are ± standard deviation.

Sequence comparison revealed that the 243-bp bidirectional minimal promoter is evolutionarily conserved among distant organisms. As illustrated in Figure 5, although neither TATA-box nor CCAAT element were found in this intergenic region, several potential transcription factor binding sites were recognized *in silico *by MatInspector  and MOTIF  softwares. These potential binding sites include two for TFAP-4 (denoted as TFAP-4(A), at nt -407 to -400; and TFAP-4(B), at nt -264 to -255), one for MZF-1 (at nt -398 to -391), one for USF (at nt -362 to -353), two for NRF-2 (denoted as NRF-2(A) at nt -307 to -298; and NRF-2(B) at nt -299 to -290) and one for YY-1 (at nt -232 to -215). Among these putative binding sequences, some core binding sites and their flanking segments such as those for USF, NRF-2, and YY-1 were found to be evolutionarily more conserved than other transcription factor binding sites (Figure 5). In particular, as the USF-binding sequence is known to be one of the most frequently predicted motifs in many gene promoters, here we chose to first examine the possible role of NRF-2 and YY-1 in the activation of *C2ORF34 *and *PREPL *transcriptions. The luciferase assays performed in U87MG cells showed that point mutations at the NRF-2(A) site (termed as -203/-445^mu_NRF-2(A) ^and -445/-203^mu_NRF-2(A)^) had no major effect on the promoter activity in either direction (Figure 6). However, mutations occurring at the NRF-2(B) binding site (denoted as -203/-445^mu_NRF-2(B) ^and -445/-203^mu_NRF-2(B)^) caused a significant reduction of the luciferase activity as compared to that of the control 243-bp bidirectional minimal promoter (~74% decrease in the *C2ORF34 *direction and ~58% decrease in the *PREPL *direction, see Figure 6). These results indicate that the second NRF-2 binding sequence (from position -299 to -290) is essential for the activation of the *PREPL-C2ORF34 *bidirectional gene pair. Moreover, mutant constructs at the YY-1 site (named as -203/-445^mu_YY-1 ^and -445/-203^mu_YY-1^) displayed a drastically reduced promoter activity toward the *PREPL *direction (~86% decrease relative to that of the control 243-bp segment) but a less severe reduction in the *C2ORF34 *direction (with only a ~37% decrease relative to that of the minimal promoter). This implies that the YY-1 site is more important than the NRF-2(B) site for *PREPL *expression. The same data also conclude that the NRF-2(B) binding site is functionally more critical than the YY-1 site in driving *C2ORF34 *transcription, as evident from mutations at the NRF-2(B) site resulting in a much decreased reporter gene activity toward the *C2ORF34 *direction (Figure 6). More significantly, double mutations at the NRF-2(B) and the YY-1 binding sites (denoted as -445/-203^mu_NRF-2(B)/YY-1 ^and -203/-445^mu_NRF-2(B)/YY-1^) further decreased the promoter activity to the basal level in both directions (Figure 6). Similar results were also observed in other cell lines including brain H4, GBM8401, and GBM8901 cell lines (Additional File 3). Together these findings provide the first strong evidence that, in addition to their individual effects on the full activity of the bidirectional minimal promoter region, the NRF-2 and YY-1 transcription factors may coordinately participate in driving both gene expressions in an additive manner.

### Characterization of the specific NRF-2 and YY-1 transcription factors binding to the bidirectional promoter by EMSA

**Figure 7:**
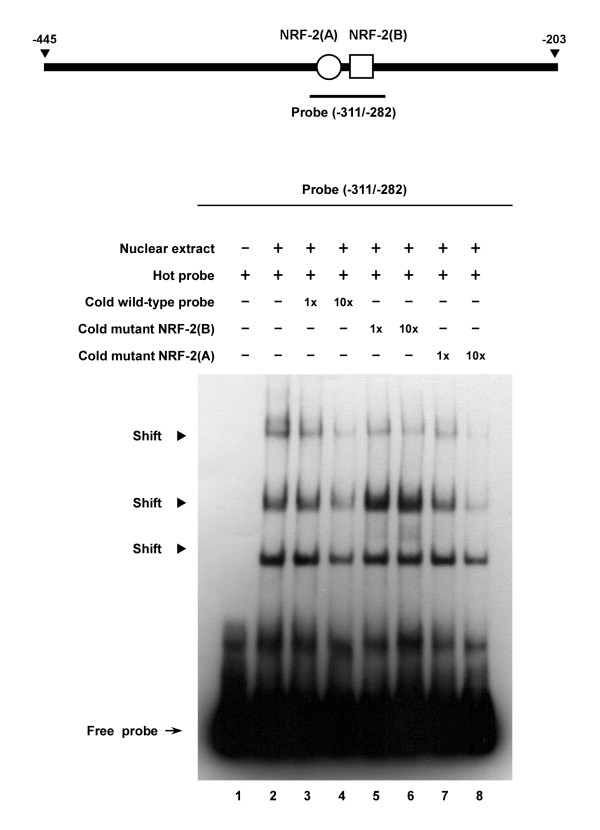
**Assessment of the effect of NRF-2 binding elements on the formation of protein/DNA complex**. The EMSA analysis was conducted as described in the Methods section. The double-stranded oligonucleotide probe (-311/-282) encompassing both NRF-2(A) and NRF-2(B) binding sites is shown on top. The arrow indicates free probe, and the arrowheads indicate three shifted bands found in the bottom panel. Lane 1 represents the biotin-labeled probe alone. Lane 2 represents the hot probe incubated with nuclear extracts. Lanes 3 and 4 represent the hot probe incubated with nuclear extracts in the presence of either 1- or 10-fold molar excess of cold wild-type probe, respectively. Lanes 5 and 6 represent the hot probe incubated with nuclear extracts in the presence of either 1- or 10-fold molar excess of unlabelled probe with mutation at the NRF-2(B) site, respectively. Lanes 7 and 8 are the same as lanes 5 and 6 except a cold probe with mutation at the NRF-2(A) site was used.

**Figure 8:**
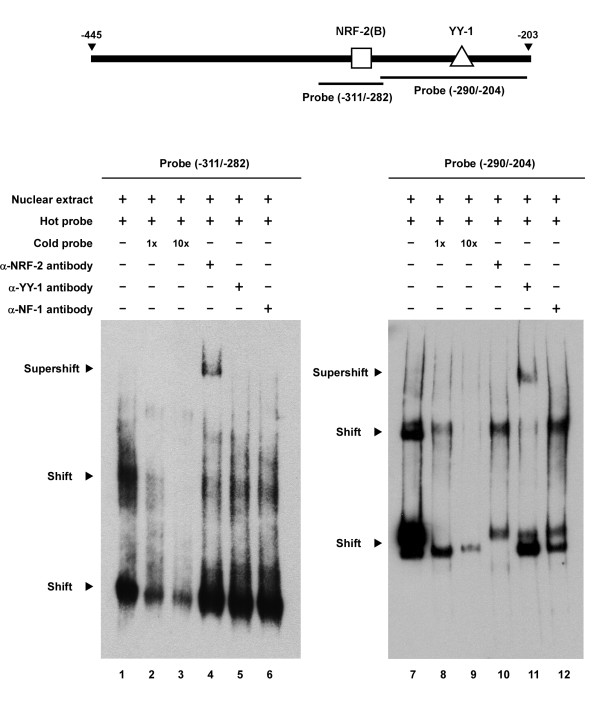
**Identification of NRF-2 and YY-1 binding specificities to the bidirectional minimal promoter**. The probe (-311/-282) which includes NRF-2 binding sites and the probe (-290/-204) which contains the sole YY-1 binding site are shown on top. Lanes 1 and 7 represent each probe incubated with nuclear extracts alone. Lanes 2 and 8 represent each probe incubated with a 1-fold molar excess of the cold competitor oligonucleotide. Lanes 3 and 9 represent each probe competed with a 10-fold molar excess of the unlabeled competitor oligonucleotide. Lanes 4 to 6 and 10 to 12 represent the same EMSA performed in the presence of three different antibodies: anti-NRF-2, anti-YY-1, and the negative control, anti-NF-1 antibody. The experiments were repeated twice.

After we assessed the regulatory importance of the NRF-2 and YY-1 binding sites within the intergenic region, the EMSA was performed to study the protein-binding to such sites. As shown in Figure 7, by incubating cell nuclear protein extracts with the -311/-282 probe that contained two NRF-2 binding sites, three major shifted bands were detected (Figure 7, lane 2). These band-shifts were sequence-specific as addition of an excess of unlabeled wild-type probe could compete out the binding (Figure 7, lanes 3 and 4). Interestingly, while competitors containing mutated NRF-2(B) site failed to affect the binding of nuclear proteins to hot probe (Figure 7, lanes 5 and 6), a strong competitive effect was obtained with the use of a 10-fold excess of cold probe with mutation at the NRF-2(A) site (Figure 7, lane 8). These results again indicate that NRF-2(B), the second NRF-2 binding sequence from position -299 to -290, is more important than NRF-2(A) in regulating the transcription of the *PREPL-C2ORF34 *bidirectional gene pair. To more clarify the specific binding of NRF-2 and YY-1 to the bidirectional promoter, the nuclear extracts were incubated with antibodies against NRF-2, YY-1, and NF-1 to examine the inhibition and/or supershift of the protein/DNA complex. As revealed in Figure 8, by incubating nuclear protein extracts either with the same -311/-282 probe or with the -290/-204 probe which contained the sole YY-1 binding site, several retarded bands were detected (Figure 8, lanes 1 and 7). Such a binding activity with the -311/-282 probe or -290/-204 probe could be significantly competed with 1- or 10-fold molar excess of unlabeled oligonucleotide (self-competition; Figure 8, lanes 2–3 and 8–9). Furthermore, when EMSA was performed in the presence of specific anti-NRF-2 or anti-YY-1 antibodies, a supershifted band was observed in each case (Figure 8, lanes 4 and 11). In both cases no supershift was observed with the negative control anti-NF-1 antibody (Figure 8, lanes 6 and 12). These results suggest that the two identified binding sites are indeed associated with sequence-specific binding of NRF-2 and YY-1 transcription factors present in the cell nuclear extracts.

### In vivo occupancy of the bidirectional minimal promoter by endogenous NRF-2 and YY-1 transcription factors

**Figure 9:**
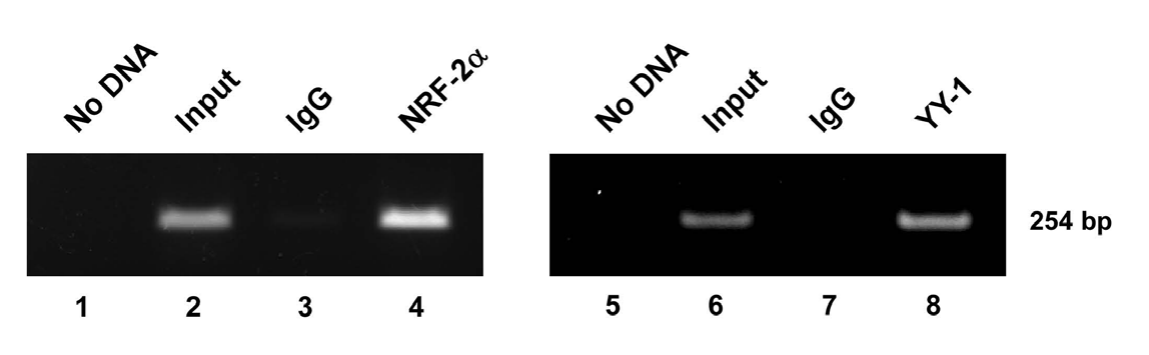
**Semi-quantitative PCR products from DNA prepared by ChIP assays**. Lanes 1 and 5: No DNA control. Lanes 2 and 6: The *Input *lanes correspond to PCR products derived from a 1/10 dilution of chromatin prior to immunoprecipitation. Lanes 3 and 7: The *IgG *lanes correspond to PCRs containing chromatin immunoprecipitated with antibodies against control IgG. Lanes 4 and 8: The *NRF-2α *and *YY-1 *lanes correspond to PCRs containing chromatin immunoprecipitated with antibodies against NRF-2α and YY-1, respectively. All the amplified products represent a 254-bp region (from position -66 to -319) containing both the NRF-2 and YY-1 binding sites of the *PREPL-C2ORF34 *bidirectional gene pair. Additional ChIP assays were performed in other cell lines with similar results (presented in Additional File 4).

To further verify whether NRF-2 and YY-1 are actually bound to the bidirectional minimal promoter of the *PREPL-C2ORF34 *gene pair *in vivo*, chromatin immunoprecipitations (ChIPs) were performed in U87MG cells using antibodies specific for NRF-2 and YY-1. As a negative control, another immunoprecipitation from the same stock was carried out using anti-IgG antibody. The regions surrounding the functional NRF-2 and YY-1 binding sites were analyzed in parallel on immunoprecipitated chromatin. For PCR amplifications, a 1/10 dilution of input chromatin was selected as a standard to indicate the efficiency of the PCR reactions. As shown in Figure 9, while nothing was detected with the use of the control anti-IgG antibody, fragments from the bidirectional minimal promoter of the intergenic region were able to be immunoprecipitated by both anti-NRF-2 and anti-YY-1 antibodies (Figure 9, lanes 4 and 8, respectively) in higher amounts than the 1/10 input chromatin (Figure 9, lanes 2 and 6). Similar results were also obtained from other ChIP assays using additional cell lines such as brain H4, GBM8401 and GBM8901 cell lines (Additional File 4). Thus, it is clear from these studies that NRF-2 and YY-1 are bound specifically to the bidirectional minimal promoter of the *PREPL-C2ORF34 *head-to-head gene pair *in vivo*.

## Discussion

Bidirectional promoters have received considerable attention because of their capability to regulate the transcription of two downstream genes [32]. They are commonly present in prokaryotes and low eukaryotes but are considered to be rare in higher organisms. Recent genome sequencing efforts, however, have revealed that a large number of mammalian genes are in fact closely arranged as divergent (head-to-head) gene pairs and separated only by a less than 1 kb of intergenic spacer [[8,10,33]; and our unpublished observations]. No definite explanation can yet be given for the biological and evolutionary advantages of this distinct type of divergent gene pairs. But theoretically, only when two genes are divergently organized in close proximity (now referred more as "bidirectional gene pairs") can be co-expressed and co-regulated by a shared intergenic bidirectional promoter [11,34]. This statement is supported by a recent study of whole-genome microarray data showing that, in a given cell, approximately 68% of divergent genes are transcribed, compared with 44% of all human genes [32]. It seems likely that the presence of bidirectional promoters in mammalian genomes is to facilitate functional co-regulation of two adjacent genes, thus making bidirectional gene pairs more favored by natural selection than any other gene organization. By analyzing and comparing the intergenic sequences of bidirectional gene pairs in different species, it has been shown that the majority of intergenic regions lack TATA and CCAAT boxes but are rich in GC content [35-42]. Those findings are consistent with our characterization of the *PREPL-C2ORF34 *bidirectional gene pair, in that neither a TATA box nor a CCAAT element could be found within the identified 405-bp intergenic region (Figure 5). A CpG-rich island was indeed recognized, but its methylation status does not seem to play an important role in regulating the expression levels of the two downstream genes (see Additional File 2). It was also reported that the intergenic regions found in mammalian genomes often display a mirror sequence composition, such that the Gs and Ts dominate on one side of the midpoint whilst Cs and As dominate on the other side [43]. In the case of the *PREPL-C2ORF34 *bidirectional gene pair presented in this work, however, no such a composition was observed.

It is of interest to note that the *PREPL-C2ORF34 *gene pair has several features in common with some other human bidirectional gene pairs such as the Parkinson's disease-related *PACRG-PARK2 *gene pair at 6q25.2–27, and the brain-disease related *PDCD10-SERPINI1 *gene pair at 3q26 [44,45]. In all three cases, the paired genes are non-homologous to each other. *PREPL*, like *PARK2 *and *SERPINI1*, has its tissue specificity and is present mainly in brain and/or heart; where as *C2ORF34*, like *PACRG *and *PDCD10*, is expressed like a housekeeping gene in all tissues (Figure 1). More significantly, the genetic variations found in these genes are often correlated with brain or genetic abnormalities. This implies that brain, being the most complex and metabolically active organ in the body, may utilize various bidirectional promoters to direct gene expressions and cellular functions in a more efficient and comprehensive manner.

*C2ORF34 *and *PREPL *are known to lie immediately adjacent to each other not only in humans but also in many other species. Their evolutionarily conserved gene configuration and close proximity indicate that both genes need to be tightly paired and may thus be co-regulated by some common transcriptional regulatory elements. By generating a series of partial deletion constructs using firefly luciferase as the reporter gene, we first identified a 273-bp GC-rich segment, from position -446 to -718, which may serve as a repressive element to suppress in particular the transcriptional activity of the *C2ORF34 *gene. This repressive effect is most likely due to some yet unidentified transcriptional repressor, or may be caused by subsequent disturbance of the chromatin structure within this GC-rich DNA region. Results from luciferase activity assays further suggest that the essential promoter activity is carried within a 243-bp (from position -445 to -203) genomic region, and that two transcription factors, NRF-2 and YY-1, can bind onto this critical bidirectional minimal promoter to regulate the expression of both *C2ORF34 *and *PREPL *genes (Figure 3, 6, 7, 8). It should be noted that the nucleotide sequence of the -718/-446 segment revealed no ATG motifs that may interfere with the downstream luciferase gene expression at the level of translation. Nonetheless, we need to point out that although highly unlikely, we cannot completely rule out the possibility that RNA splice sites may have bypassed the ATG of the luciferase reporter in our analysis.

NRF-2, also termed as GA-binding protein or GABP, has long been known to act as a key nuclear activator in the regulation of many mitochondrial-related genes [46]. Similar to a previous study on the promoter region of the outer mitochondrial membrane translocase *TOMM20 *gene [47], in our identified 405-bp intergenic fragment only one of the two putative NRF-2 binding sites was found responsible for driving the bidirectional promoter activity. More significantly, NRF-2 itself is also arranged as a bidirectional gene pair with its adjacent *ATP5J *(an ATP synthase coupling factor) gene on human chromosome 21. By binding to its 5'-intergenic region shared with the *ATP5J *gene, NRF-2 is able to auto-regulate its own gene transcription and subsequently affect the expression levels of downstream mitochondrial-related genes [48]. In the case of the *PREPL-C2ORF34 *gene pair, because NRF-2 is shown to be more important in driving *C2ORF34 *expression and because patients with the 2p21 deletion syndromes (in particular those occurring at *C2ORF34*) often exhibit a unique mitochondrial dysfunction [19,22], we speculate that the ubiquitously expressed *C2ORF34 *gene may possess some cellular functions related to mitochondrial metabolisms and be ultimately regulated by NRF-2 along with the collaboration of YY-1 transcription factor.

Previously, YY-1 had been reported to serve as a necessary and sufficient activator for the expression of some bidirectional gene pairs [13,49]. As for the evolutionarily conserved *PREPL-C2ORF34 *gene pair, mutations occurring at the YY-1 binding site were shown to cause significantly decreased promoter activity toward the *PREPL *direction (Figure 6). This suggests that YY-1 is functionally more important than NRF-2 in driving *PREPL *transcription. Moreover, because the dual activator/repressor role of YY-1 is often related to its cellular concentration as well as its ability to interact with other cofactors [50-52], here we propose that YY-1 can directly bind to the bidirectional minimal promoter of the *PREPL-C2ORF34 *gene pair and, depending on the intracellular concentrations of YY-1 and its interacting cofactors such as NRF-2, serve as both a positive regulator and a negative regulator in determining the tissue distribution pattern of the *PREPL *gene. Finally, double mutations at both the NRF-2 and YY-1 binding sites completely abolished the activity of the bidirectional minimal promoter in both directions (Figure 6). This implies that, in addition to their individual effect on the full activity of the bidirectional minimal promoter region, NRF-2 and YY-1 coordinately participate in driving both *C2ORF34 *and *PREPL *gene expressions in an additive manner. The functional cooperation between these two transcription factors, along with their binding efficacy to the asymmetric bidirectional minimal promoter and some cis-acting repressive elements, is essential for the transcriptional activation and tissue distribution of the *PREPL-C2ORF34 *head-to-head gene pair.

## Conclusion

Unlike previous reports suggesting that two adjacent genes in a head-to-head configuration can be co-regulated by a single transcription factor binding to a specific promoter segment, here we demonstrated that the expression of the disease-related *PREPL-C2ORF34 *bidirectional gene pair is in fact dependent on the synergic cooperation of two transcription factors interacting with a 405-bp intergenic DNA region. Although *PREPL *is present primarily in brain and heart, *C2ORF34 *is ubiquitously and abundantly expressed in almost all tissues examined. Their distinct expression patterns may not be directly regulated by DNA methylation but rather be controlled by the coordinated binding of NRF-2 and YY-1 onto a 243-bp asymmetric bidirectional minimal promoter. An additional GC-rich region, from nt -446 to nt -718, is likely to serve as a repressive element suppressing the transcription activity of the *C2ORF34 *gene. Finally, various analyses including luciferase activity experiments, electrophoretic mobility shift assays and *in vivo *chromatin immunoprecipitation verified the direct binding of NRF-2 and YY-1 onto the shared intergenic region, thus confirming that these two transcription factors function coordinately with the bidirectional minimal promoter to regulate the transcriptions of both *PREPL *and *C2ORF34 *genes.

## Methods

### Cell cultures

All culture media were purchased from Gibco Invitrogen (Carlsbad, CA, USA). Human neuroglioma U87MG, glioblastoma GBM8401 and GBM8901, and lung fibroblast IMR90 and WI38 cells were obtained from the American Type Culture Collection (ATCC, Manassas, VA, USA) and grown in Minimum Essential Medium Eagle (MEM-E, high glucose with L-glutamine and phenol red) supplemented with 10% fetal bovine serum (FBS) and pyruvate (HyClone, Logan, UT, USA). Human neuroblastoma H4 cells were also purchased from ATCC but cultured in Dulbecco's Modified Eagle Medium (D-MEM) supplemented with 10% FBS and 0.1 mM non-essential amino acids (Biological Industries, Kibbutz Beit Haemek, Israel). All cell lines were maintained at 37°C in a humidified atmosphere of 5% CO_2 _in air.

### Northern blot analysis

Human 12 major tissue polyA+ blot (HB-2010) was purchased from OriGene (Rockville, MD, USA). To increase the probe specificity and sensitivity in the recognition of all possible splicing variants, a 735-bp probe spanning from exon 9 to exon 14 of the *PREPL *gene was amplified from FirstChoice PCR-Ready human brain cDNA (Ambion, Austin, TX, USA) using the forward primer 5'-ATGGATTTGAAAATGAATTTCAGG-3' and the reverse primer 5'-TCAGAATTTCAGGTATTTCTTAAGA-3'. Another 498-bp probe, spanning from exon 1 to exon 6 of the *C2ORF34 *gene, was amplified from the same cDNA using the forward primer 5'-ATGGAGTCGCGAGTCGCGGACGCTG-3' and the reverse primer 5'-AGCAACCATGAGCCCAGCCAAGCA-3'. All the PCRs were performed on T3 thermocycler (Whatman Biometra, Goettingen, Germany) using Ex Taq polymerase (Takara, Otsu, Singa, Japan) under the following condition: 1 cycle of 95°C for 10 min; 30 cycles of 95°C for 30 sec, 60°C for 30 sec, and 72°C for 40 sec; and 1 cycle of 72°C for 10 min. All amplified PCR products were visualized on agarose gels and confirmed by autosequencing on an ABI PRISM 3700 automated sequencer (Applied Biosystems, Foster City, CA, USA) by the core facility of the National Health Research Institutes (Zhunan, Taiwan). The sequence-verified probes, along with the internal control β-actin (*ACTB*) probe supplied by the manufacturer (OriGene), were labeled with [^32^P]dCTP by random prime labeling system (Amersham/GE Healthcare, Piscataway, NJ, USA). Pre-hybridization and hybridization of the membrane was performed in ULTRAhyb solution (Ambion) at 42°C. The hybridized membrane was washed twice with 2× SSC and 0.1% (w/v) SDS for 20 min at 42°C followed by washed twice with 0.25× SSC and 0.1% (w/v) SDS (w/v) for 20 min at 65°C. The hybridization signal was detected by exposure to X-ray film (Eastman Kodak, Rochester, NY, USA). The signal was stripped off and rehybridized with *ACTB *as an internal control.

### 5'-Rapid amplification of cDNA ends (RACE)

The 5'-RACE analysis on the human fetal brain was carried out by two consecutive PCR reactions with the use of First Choice RNA ligase-mediated RACE kit (Ambion). In the first round of PCR, at least five reverse primers were attempted for each gene in a trial-and-error fashion until successful PCR amplification occurred. In brief, the PCR was performed using a forward 5'-RACE outer primer provided by the manufacturer and the most appropriated and specific reverse primer for *PREPL *(5'-CAATCAATGAAGGGCTGGTCTAACT-3') or for *C2ORF34 *(5'-GGTCCAGAAACAGGCAGTCAGCACACATAACA-3') under the following conditions: 1 cycle at 95°C for 5 min; 40 cycles at 95°C for 1 min, 55°C for 1 min and 72°C for 1 min; and 1 cycle at 72°C for 10 min. For the second round of PCR, the DNA amplified during the first round of PCR was diluted 1:100 and reamplified using a forward 5'-RACE inner primer provided by the manufacturer with either a reverse inner *PREPL *primer (5'-CCAGTGAAGGCAGGCCGGAGAAC-3') or *C2ORF34 *primer (5'-GGTCCAGAAACAGGCAGTCAGCACACATAACA-3') under the following conditions: 1 cycle at 95°C for 5 min; 40 cycles at 95°C for 1 min, 57°C for 1 min and 72°C for 1 min; and 1 cycle at 72°C for 10 min. The desired PCR products were isolated from agarose gels and cloned into a pGEM-T Easy vector (Promega, Madison, WI, USA) followed by transformation into competent Top10 *E. coli *cells. Two to three independent clones were then selected, purified, and sequenced with generic sequencing primer to determine the 5'-ends of *C2ORF34 *and *PREPL *transcripts.

### Interspecies comparison of the PREPL and C2ORF34 genes

The comparisons were performed based on the data obtained from genome assemblies of the human NCBI Build 36.2, the mouse NCBI Build 37.1, the rat RGSC 3.4, the dog NCBI Build 2.1, and the chicken NCBI Build 2.1 databases . In brief, for all the genes (i.e. *C2ORF34*, *PREPL*, *SLC3A1 *and *PPM1B*) that were found to be clustered on human chromosome 2p21, at least three features were examined and compared in each genome. These features included chromosomal localization, orientation, and distance between the two adjacent genes. All nucleotide sequences, in particular those for *C2ORF34 *and *PREPL*, were downloaded from the NCBI databases and aligned using Vector NTI software (InforMax, Bethesda, MD, USA).

### Bisulfite sequencing analysis of DNA methylation

Genomic DNAs used for bisulfate analysis were purchased from Biochain (Biochain Institute, Hayward, CA, USA) including samples of normal brain (Lot #A712158: male, 27-year old), normal colon (Lot #A80504: male, 23-year old) and normal testis (Lot #A905081: male, 26-year old) tissues. The CpG methylation status was analyzed using the Zymo EZ DNA Methylation-Gold Kit (Zymo Research, Orange, CA, USA) according the manufacturer's instructions. In brief, 1 μg of each genomic DNA was treated with the CT Conversion reagent supplied within this kit under the following conditions: 98°C for 10 min, 64°C for 4 h, and then at 4°C until use. All bisulfite-converted DNA samples were purified by the Zymo-Spin IC column with 600 μl M-Binding buffer, 100 μl M-Wash buffer, 200 μl M-Desulphonation buffer, 200 μl M-Wash buffer, and then eluted with 10 μl M-Elution buffer. The samples were then PCR-amplified using the forward primer (5'-CCACATAATTCCTCCTCTT-3') and reverse primer (5'-TCCACATCTCTCTCATCCTCTTTTC-3') under following condition: 1 cycle of 95°C for 10 min; 30 cycles of 95°C for 30 sec, 50°C for 30 sec, and 72°C for 40 sec; and 1 cycle of 72°C for 10 min. The amplified products were cloned into a pGEM-T Easy vector (Promega) followed by transformation into competent Top10 *E. coli *cells. Four individual clones for each tissue sample were randomly selected and subjected to sequencing by the core facility of the National Health Research Institutes.

### Construction of reporter gene plasmids

All restriction enzymes were purchased from Takara. The genomic DNA of peripheral blood lymphocytes was isolated from a healthy donor using the QIAamp Blood kit (Qiagen, Hilden, Germany) as described by the manufacturer. Various intergenic fragments between *C2ORF34 *and *PREPL *were amplified by PCR using ExTag polymerase (TaKaRa) with a series of forward and reversed primers (listed in Table 1) containing a BglII or HindIII cutting site, respectively. Each amplified fragment was digested with BglII and HindIII and then cloned into pGL3-Basic vector (Promega) followed by autosequencing to ensure no mutations had been introduced during the amplification reaction. In order to facilitate subsequent characterization of the promoter activity of the intergenic region, the positions of the most 5' exon of *PREPL *(exon 1a) or *C2ORF34 *(exon 1) are numbered as +1 and -406, respectively. Thus, the first intergenic nucleotide in front of the *PREPL *transcriptional start site is numbered as -1, and the intergenic nucleotide right before the transcriptional start site of *C2ORF34 *is numbered as -405.

**Table 1:** List of primers used to amplify the *PREPL-C2ORF34 *bidirectional promoter fragments

Constructs*	**Primer sequence**	
***C2ORF34***	**(Forward)**	**(Reverse)**

+335/-718	5'-GAAGATCTGAATGCAACAGGGAG-3'	5'-GAAGCTTCGCAGGTGGCTCCCGG-3'
-11/-718	5'-AGATCTAGCACGAGCAACAGGCAGAT-3'	5'-GAAGCTTCGCAGGTGGCTCCCGG-3'
-203/-718	5'-AGATCTGGTCCGCCCTCACTCAAGAT-3'	5'-GAAGCTTCGCAGGTGGCTCCCGG-3'
-420/-718	5'-AGATCTCGGGTGTGGAAGGCTCCAGTGAGAT-3'	5'-GAAGCTTCGCAGGTGGCTCCCGG-3'
-580/-718	5'-AGATCTGGTAAGGGAGAACCTGCTCG-3'	5'-GAAGCTTCGCAGGTGGCTCCCGG-3'
-203/-445	5'-AGATCTGGTCCGCCCTCACTCAAGAT-3'	5'-AAGCTTATCTCACTGGAGCCTTCCACACCCG-3'
-262/-445	5'-AGATCTTCGGCCCTGGTTGCCAAGGAGAT-3'	5'-AAGCTTATCTCACTGGAGCCTTCCACACCCG-3'
-203/-385	5'-AGATCTGGTCCGCCCTCACTCAAGAT-3'	5'-AAGCTTCCTGCCAACTTCTTCCCTGT-3'
-203/-345	5'-AGATCTGGTCCGCCCTCACTCAAGAT-3'	5'-AAGCTTACCCTCCCTCCACCGGACCA-3'
		
***PREPL***	**(Forward)**	**(Reverse)**

-718/+335	5'-AGATCTTCGCAGGTGGCTCCCGGC-3'	5'-AAGCTTGATCTGAATGCAACAGGGAG-3'
-600/+335	5'-AGATCTCGAGCAGGTTCTCCCTTACC-3'	5'-AAGCTTGATCTGAATGCAACAGGGAG-3'
-445/+335	5'-AGATCTCACTGGAGCCTTCCACACCCG-3'	5'-AAGCTTGATCTGAATGCAACAGGGAG-3'
-225/+335	5'-AGATCTTGAGTGAGGGCGGACCAGA-3'	5'-AAGCTTGATCTGAATGCAACAGGGAG-3'
-33/+335	5'-AGATCTGCCTGTTGCTCGTGCTAGA-3'	5'-AAGCTTGATCTGAATGCAACAGGGAG-3'
-445/-203	5'-GAAAGATCTCACTGGAGCCTTCCACACCCG-3'	5'-AAGCTTCTGGTCCGCCCTCACTCAAGAT-3'
-445/-262	5'-GAAAGATCTCACTGGAGCCTTCCACACCCG-3'	5'-AAGCTTCGGCCCTGGTTGCCAAGGAGAT-3'
-385/-203	5'-AGATCTCCTGCCAACTTCTTCCCTGT-3'	5'-AAGCTTCTGGTCCGCCCTCACTCAAGAT-3'
-345/-203	5'-AGATCTACCCTCCCTCCACCGGACCA-3	5'-AAGCTTCTGGTCCGCCCTCACTCAAGAT-3'

*All construct names refer to the positions of nucleotides, counting from the first to the last nucleotide of the inserted genomic DNA sequences. The inserted fragments were first amplified by PCR using a series of forward primers with a BglII site (5'-AGATCT) and reversed primers with a HindIII site (5'-AAGCTT). Each amplified fragment was then digested with two restriction enzymes and cloned into the pGL3-Basic vector (Promega).

### Transient transfection and dual luciferase reporter assay

For luciferase reporter assays, the cells from different cell lines were first seeded in 12-well culture plates at a density of 1 × 10^5 ^cells per well and allowed to grow overnight. Cells were then co-transfected, in triplicate, with 3 μl of Lipofectamine 2000 (Gibco Invitrogen) and a mixture of 525 ng of DNA consisting of 500 ng of the tested pGL3 promoter constructs and 25 ng of the pRL-TK *Renilla *luciferase reporter plasmid (Promega) in a serum-free medium. The pGL3-promoter plasmid containing the SV40 promoter was used as the positive control. After a 2-h transfection at 37°C, the medium was replaced with fresh culture medium supplemented with 10% FBS and cells were incubated for another 24 h. After incubation, cells were harvested and lysed in Passive Lysis Buffer (Promega) at 4°C for 30 min. Cellular debris was removed after centrifugation at 10,000 × g for 5 min at 4°C. The chemiluminescence of the two different luciferases was measured using a SIRIUS luminometer (Berthold, Bad Wildbad, Germany). The transfection efficiency and cell viability were normalized with the pRL-TK plasmid containing the *Renilla *luciferase gene under the control of the TK promoter. Relative luciferase activity of each clone was normalized to the pGL3-promoter vector containing a firefly luciferase gene driven by the weak SV40 promoter. The experiments were performed in triplicate and repeated independently at least three times.

### Preparation of nuclear proteins from cultured cells

Nuclear extracts were prepared from cultured U87MG and H4 cells using nuclear extraction kit (Panomics, Fremont CA, USA) according to the manufacturer's specifications. Cells grown to 80–90% confluence in 10 cm culture plates were washed twice with an appropriate volume of ice-cold PBS. After complete removal of PBS by centrifugation, cells were treated with 1 mL of Buffer A working reagent containing 1 mM DTT, 10 μl protease inhibitor, 10 μl phosphatase inhibitor I, and 10 μl phosphatase inhibitor II for 10 min at 4°C. Cells were released from the bottom of the culture plate by using a sterile cell scraper and transferred into a 1.5 mL microcentrifuge tube with 14,000 × g for 5 min at 4°C. The remnant pellet was re-suspended with 100 μl Buffer B working reagent containing 1 mM DTT, 1 μl protease inhibitor, 1 μl phosphatase inhibitor I, and 1 μl phosphatase inhibitor II on ice for 60 min with vortex every 10 min. Nuclear proteins were subsequently obtained by centrifugation at 14,000 × g for 5 min at 4°C.

### Electrophoretic Mobility Shift Assays (EMSA)

Electrophoretic mobility shift assays were performed by using the EMSA gel shift kit (Panomics) according to the manufacturer's instructions. In brief, the EMSA probe for YY-1 binding assay was synthesized by PCR reaction using the forward primer (5'-ACTCGCATCTCCTTGGCAACCAGGG-3') and the reverse primer (5'-GGCCCTGGTTGCCAAGGAGATGCG-3') under the following conditions: 1 cycle at 95°C for 5 min; 30 cycles at 95°C for 30 sec, 68°C for 30 sec, and 72°C for 20 sec; and 1 cycle at 72°C for 10 min. The EMSA probe for NRF-2 binding analysis was synthesized with the forward primer (5'-CCCGAATTCCGCATTTCCGGTACTCGCATC-3') and the reverse primer (5'-GATGCGAGTACCGGAAATGCGGAATTCGGG-3') under the following conditions: 1 cycle at 95°C for 5 min, 70°C for 30 sec, and 60°C for 30 sec. With the same PCR condition, the mutant probe of NRF-2(A) site was generated using the forward primer (5'-CCCGAATTAAGCATTTCCGGTACTCGCATC-3') and the reversed primer (5'-GATGCGAGTACCGGAAATGCTTAATTCGGG-3'), and the mutant probe of NRF-2(B) was synthesized with forward primer (5'-CCCGAATTCCGCACTTAATGTACTCGCATC-3') and reversed primer (5'-GATGCGAGTACATTAAGTGCGGAATTCGGG-3'). The specific hot probes were prepared by labeling the same oligonucleotide probes with biotin. The DNA-protein binding reaction was performed in supplemented binding buffer containing 1 μl of probe, 5 μg of nuclear extracts, and 1 μl poly(dI-dC) at 15°C for 30 min. For competition studies, the nuclear extracts were incubated with 1- or 10-fold molar excess of unlabelled competitor oligonucleotides. For supershift analysis, the same amounts of nuclear extracts were preincubated with 2 μl of either anti-NRF-2 (sc-28311 X; Santa Cruz Biotechnology, Santa Cruz, CA, USA), anti-YY-1 (sc-1703 X; Santa Cruz), or anti-NF-1 (sc-870 X; Santa Cruz) antibody at 15°C for 15 min prior to the binding of the probe. All the DNA-protein complexes were analyzed on native 6% (w/v) polyacrylamide gels in 0.5× TBE buffer (22.5 mM Tris-borate, 5 mM EDTA) at 5 mA for 7 to 12 h depending on the fragment size. The gels were then transferred onto nylon membranes, detected using streptavidin-HRP with a chemiluminescent substrate, and visualized by exposure to Kodak Biomax MR films.

### Semi-quantitative reverse transcription polymerase chain reaction (RT-PCR)

Total RNAs were extracted from various cell lines using TRIZOL reagent (Gibco Invitrogen) followed by DNase (Ambion) treatment to eliminate potential genomic DNA contamination. The quality and integrity of the RNAs were examined by agarose gel electrophoresis. Complementary DNA (cDNA) was synthesized from 1 μg of total RNA using oligo d(T)-anchor primer (Roche Molecular Biochemicals, Indianapolis, IN, USA) and M-MLV reverse transcriptase (Ambion) according to the manufacturer's instructions. After a 2-h incubation at 42°C, the reaction mixtures were heat inactivated at 70°C for 10 min and then applied as templates to amplify cDNAs specific for *PREPL *(amplified with the forward primer 5'-GCACGCTGATGGCCGCCTAACTAAA-3' and the reverse primer 5'-CCGCGATGGCTTCCTTGAGTTTCT-3' at 1 cycle of 95°C for 5 min; 35 cycles of 95°C for 30 sec, 64°C for 30 sec, and 72°C for 30 sec; and then 1 cycle of 72°C for 10 min). The *C2ORF34 *gene was examined with the forward primer (5'-CGCCGAGGGAATACTTTAAACCAG-3') and reverse primer (5'-GGGCAGGACGAGCAACAAGCAGAGT-3') under following condition 1 cycle at 95°C for 5 min; 30 cycles at 95°C for 30 sec, 67.5°C for 30 sec, and 72°C for 30 sec; and 1 cycle at 72°C for 10 min. *ACTB *(β-actin) serving as internal control was amplified using the primer set 5'-GATGATGATATCGCCGCGCT-3' and 5'-TGGGTCATCTTCTCGCGGTT-3' under the following conditions: 1 cycle at 95°C for 5 min; 28 cycles at 95°C for 30 sec, 64°C for 30 sec, and 72°C for 30 sec; and then 1 cycle at 72°C for 10 min.

### Chromatin immunoprecipitation assay (ChIP)

The ChIP assays were performed using the EZ ChIP kit (Upstate, Lake Placid, NY, USA) according to the manufacturer's instructions. For each assay, 3 × 10^7 ^cells from each cell line were incubated with 1% formaldehyde fixation solution for 10 min at room temperature and then quenched with glycine. The nuclei were fully resuspended in 1 mL lysis buffer with 5 μl protease inhibitor cocktail and 1 μl PMSF. The lysates were sonicated 3 times (15 min max power, 30 sec on and 30 sec off) at 4°C using Bioruptor (Diagenode SA, Liège, Belgium). Following sonication, the chromatin fragments of 200~1,000 bp with a major size of 500 bp were yielded. The sonicated chromatin was pre-cleaned with protein G agarose beads with gentle agitation at 4°C for 2 h followed by centrifugation at 5,000 × g for 1 min. One-tenth volume of the sample was diluted in a ChIP dilution buffer (1.67 mM Tris-HCl pH 8.1, 167 mM NaCl, 1.1% Triton X-100, 0.01% sodium deoxycholate, 1.2 mM EDTA) and stored at 4°C as the input fraction; the remaining solutions were divided into several aliquots and incubated with 5 μg each of specific antibodies including anti-NRF-2 (sc-28311 X; Santa Cruz Biotechnology, Santa Cruz, CA, USA), anti-YY-1 (sc-1703 X; Santa Cruze), anti-histone 3 (Upstate), and control rabbit anti-IgG (Upstate) antibodies. The protein-DNA-antibody complexes were incubated at 4°C for overnight with gentle rotation and then immunoprecipitated by protein G agarose slurry for 2 h at 4°C. The chromatin immune complexes were washed sequentially with 1 mL of low-salt buffer, high-salt buffer, LiCl buffer, and TE buffer before being eluted twice with 100 μl of elution buffer (1% SDS and 0.1 M NaHCO3). The cross-linked antibody-TF-DNA complexes were reversed by the addition of 8 μl of 5 M NaCl and heated at 65°C overnight. After removal of RNA and protein by the treatments with RNase A and proteinase K, the remnant DNA and input control were purified using the EZ ChIP kit. The precipitated DNAs were subjected to PCR in attempt to amplify the NRF-2 and YY-1 binding elements under the following conditions: 1 cycle of 95°C for 5 min; 40 cycles of 95°C for 30 sec, 68°C for 30 sec, and 72°C for 20 sec; and 1 cycle of 72°C for 5 min with the forward primer (5'-CGCGCTCTCCCCGAATT-3') and reverse primer (5'-TTGGGTTCCTGGGTTCTTTAGTC-3'). The amplified fragments were then resolved electrophoretically on a 2% (w/v) agarose gel and verified by DNA sequencing.

## List of abbreviations

PREPL: prolyl oligopeptidase-like; C2ORF34: chromosome 2 open reading frame 34; FBS: fetal bovine serum; RACE: rapid amplification of cDNA ends; TSSs: transcriptional start sites; RT-PCR: reverse transcription-polymerase chain reaction; ChIP: chromatin immunoprecipitation assay.

## Authors' contributions

CCH designed and performed the experiments and analyzed the results. WSWC supervised the project and oversaw the experiments. Both authors read and approved the final manuscript.

## Supplementary Material

Additional file 1 **Verification of the newly identified *PREPL *splice variant**. The genomic organization and transcribed mRNA of *PREPL *are illustrated in the upper panel, with each exon numbered in bold. The designed primers (indicated by arrows) for RT-PCR include the common forward primer (Exon 1a-F) derived from the alternative exon 1a of *PREPL*, and a set of reversed primers (Exons 4-R, 6-R, 8-R, 10-R, 13-R and 14-R) derived from exons 4, 6, 8, 10, 13 and 14, respectively. Bottom left: the amplified PCR products were detected on a 1.5% (w/v) agarose gel confirming the presence of the identified *PREPL *splice variant. Bottom right: the nucleotide sequences of the primers. Click here for file

Additional file 2 **CpG methylation pattern of the *PREPL-C2ORF34 *bidirectional gene pair in normal tissues**. Upper panel: The GC content from position -1,100 to +636 is calculated using Methprimer software with a 200-bp sliding window, and with a ratio of observed versus expected CpGs greater than 0.7 with an average GC content larger than 60%. The identified 322-bp CpG island (from position -286 to -607) is indicated in light blue. Lower panel: The bisulfite-treated genomic DNAs from human normal brain, colon, and testis tissues were PCR-amplified and then cloned into pGEM-T vector for autosequencing. A total of 4 clones were selected and the methylation patterns were analyzed and presented. Each row indicates an individual sequencing clone and each vertical line represents the same CpG position. The empty circles denote the unmethylated CpG sites, and the filled circles indicate the methylated CpG sites. Click here for file

Additional file 3 **Identification of the functional importance of the NRF-2 and YY-1 binding sites in the bidirectional minimal promoter**. These additional experiments were the same as for those given in Figure 6 except for using three different cell lines: brain H4, GBM8401, and GBM8901 cells. Click here for file

Additional file 4 **Semi-quantitative PCR products from DNA prepared by ChIP assays**. These additional experiments were the same as for those given in Figure 8 except for using three different cell lines: brain H4, GBM8401, and GBM8901 cells. Click here for file
