# Supplementary figures and images for: Cooperation between NRF-2 and YY-1 transcription factors is essential for triggering the expression of the PREPL-C2ORF34 bidirectional gene pair
Source: BMC Mol Biol. 2009 Jul 3;10:67. doi: 10.1186/1471-2199-10-67 (PMC2713978; doi:10.1186/1471-2199-10-67)

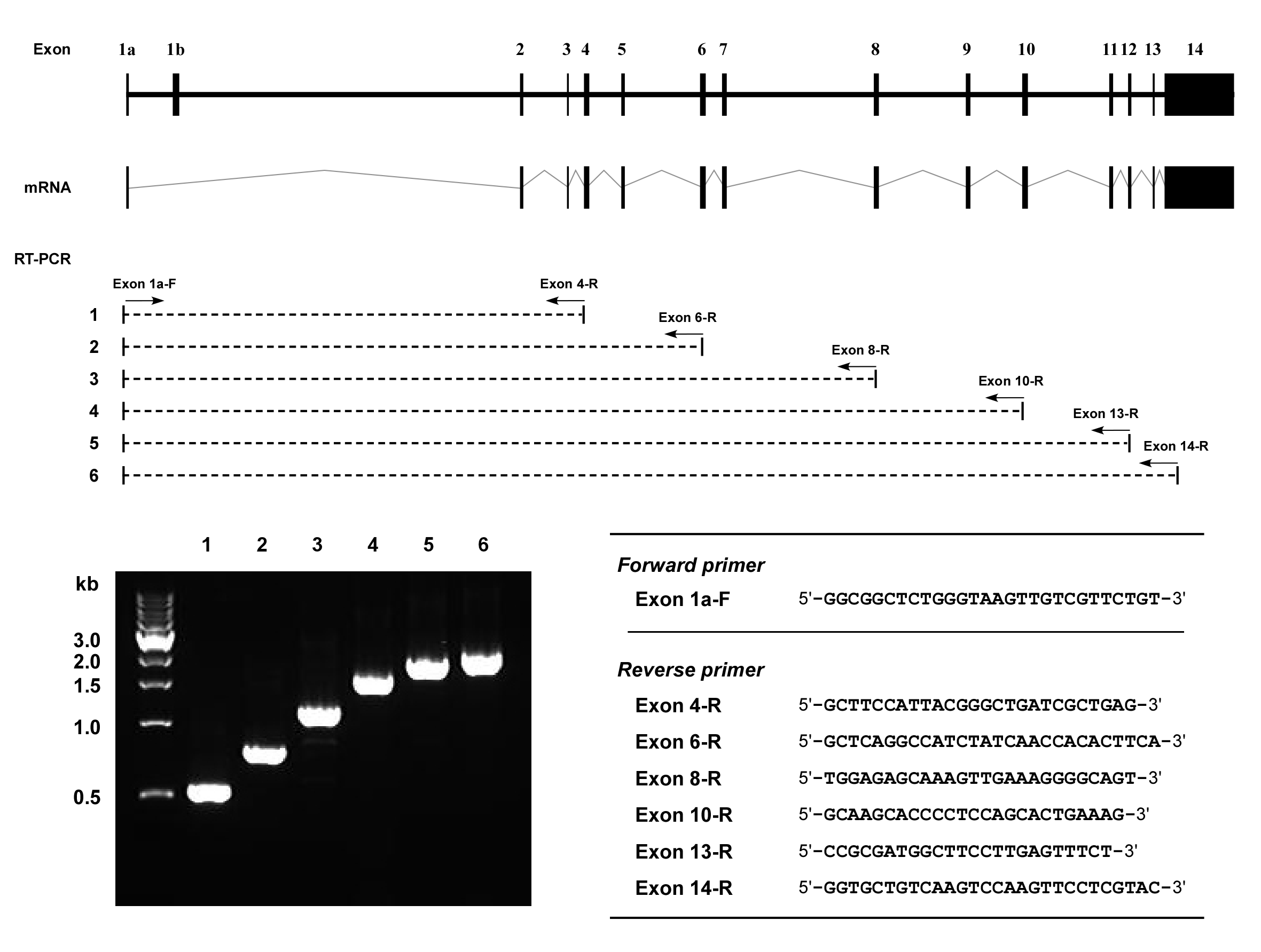

Supplement: Additional file 1 — Verification of the newly identified PREPL splice variant. The genomic organization and transcribed mRNA of PREPL are illustrated in the upper panel, with each exon numbered in bold. The designed primers (indicated by arrows) for RT-PCR include the common forward primer (Exon 1a-F) derived from the alternative exon 1a of PREPL, and a set of reversed primers (Exons 4-R, 6-R, 8-R, 10-R, 13-R and 14-R) derived from exons 4, 6, 8, 10, 13 and 14, respectively. Bottom left: the amplified PCR products were detected on a 1.5% (w/v) agarose gel confirming the presence of the identified PREPL splice variant. Bottom right: the nucleotide sequences of the primers. [file 1471-2199-10-67-S1.tiff]

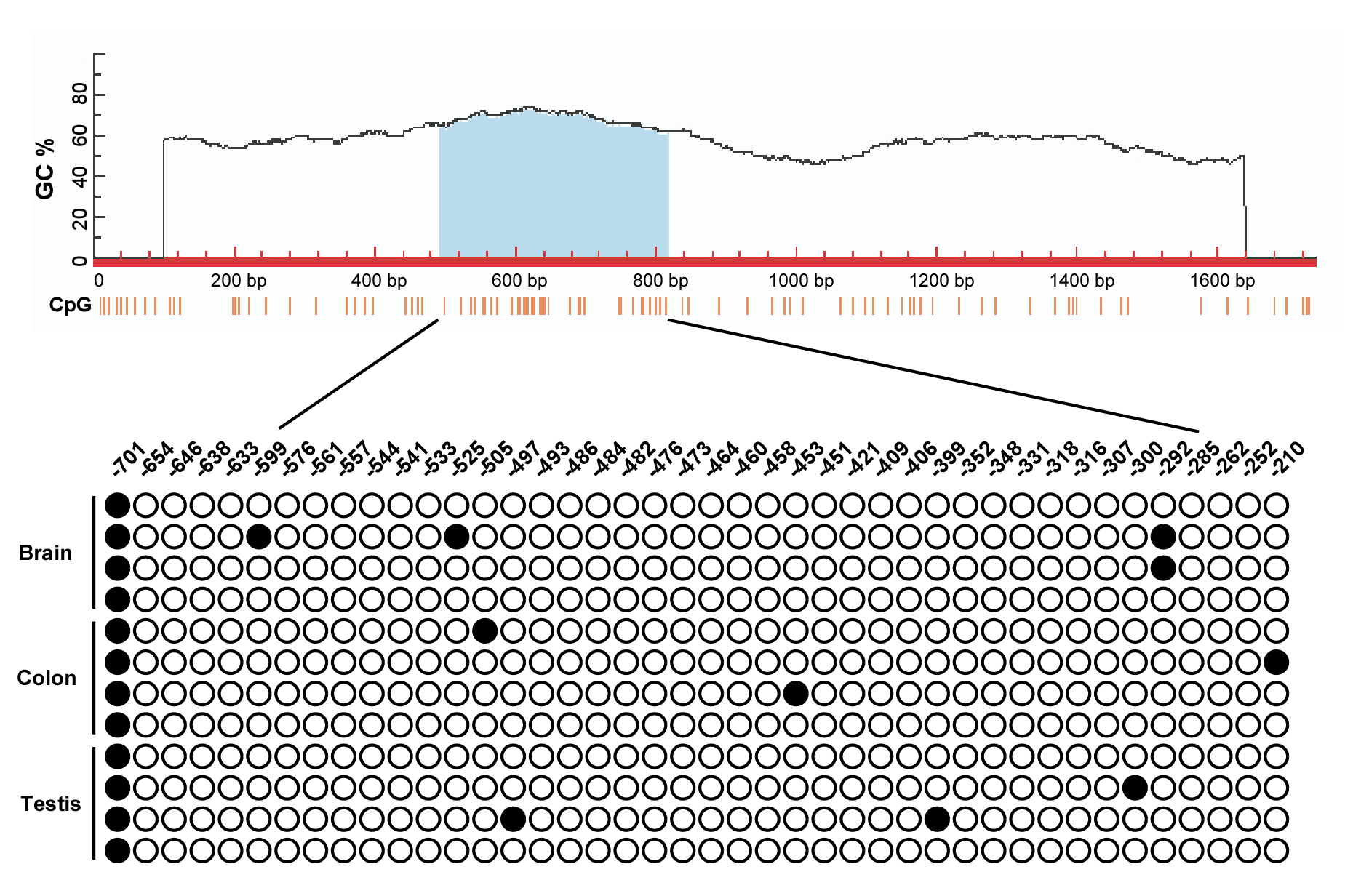

Supplement: Additional file 2 — CpG methylation pattern of the PREPL-C2ORF34 bidirectional gene pair in normal tissues. Upper panel: The GC content from position -1,100 to +636 is calculated using Methprimer software with a 200-bp sliding window, and with a ratio of observed versus expected CpGs greater than 0.7 with an average GC content larger than 60%. The identified 322-bp CpG island (from position -286 to -607) is indicated in light blue. Lower panel: The bisulfite-treated genomic DNAs from human normal brain, colon, and testis tissues were PCR-amplified and then cloned into pGEM-T vector for autosequencing. A total of 4 clones were selected and the methylation patterns were analyzed and presented. Each row indicates an individual sequencing clone and each vertical line represents the same CpG position. The empty circles denote the unmethylated CpG sites, and the filled circles indicate the methylated CpG sites. [file 1471-2199-10-67-S2.tiff]

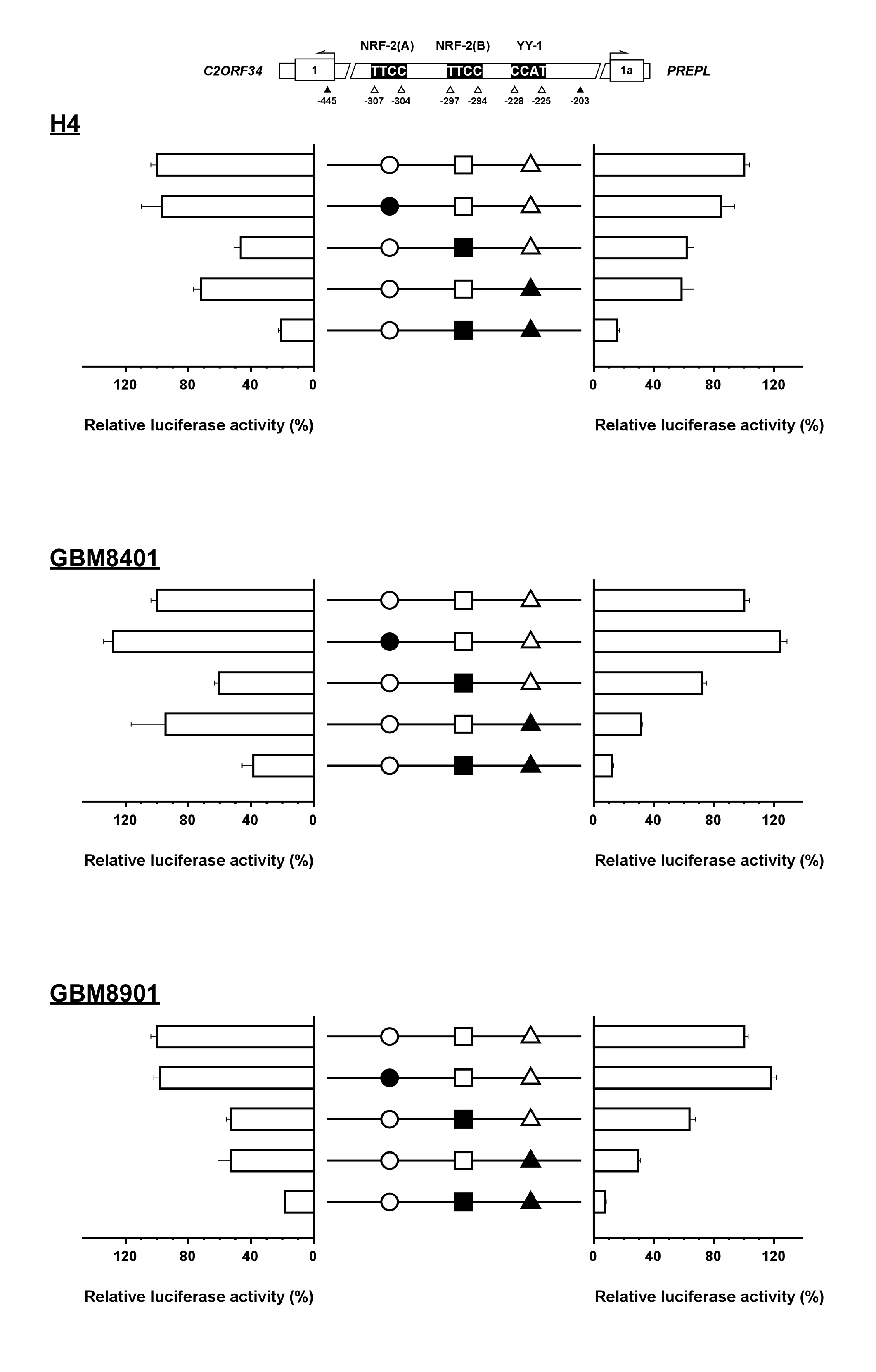

Supplement: Additional file 3 — Identification of the functional importance of the NRF-2 and YY-1 binding sites in the bidirectional minimal promoter. These additional experiments were the same as for those given in Figure 6 except for using three different cell lines: brain H4, GBM8401, and GBM8901 cells. [file 1471-2199-10-67-S3.tiff]

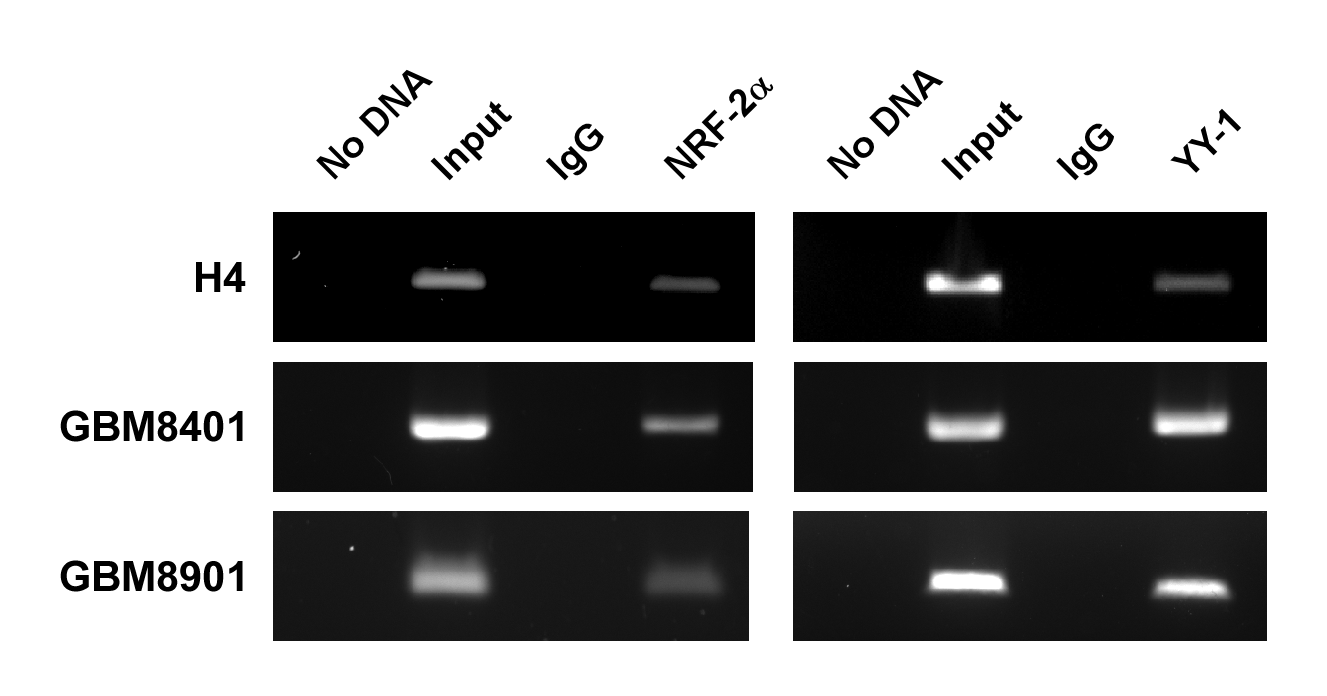

Supplement: Additional file 4 — Semi-quantitative PCR products from DNA prepared by ChIP assays. These additional experiments were the same as for those given in Figure 8 except for using three different cell lines: brain H4, GBM8401, and GBM8901 cells. [file 1471-2199-10-67-S4.tiff]
